# Gypenosides Prevent and Dissolve Cholesterol Gallstones by Modulating the Homeostasis of Cholesterol and Bile Acids

**DOI:** 10.3389/fmed.2022.818144

**Published:** 2022-04-04

**Authors:** Qian Zhuang, Jinnian Cheng, Jie Xia, Min Ning, Shan Wu, Shuang Shen, Yan Shi, Dan Huang, Zhixia Dong, Xinjian Wan

**Affiliations:** Digestive Endoscopic Center, Shanghai Jiao Tong University Affiliated Sixth People's Hospital, Shanghai, China

**Keywords:** Gypenosides, gallstones, prevention, dissolution, cholesterol, bile acids

## Abstract

Gypenosides (GPs), obtained from *Gynostemma pentaphyllum* (Thunb.) Makino, have been traditionally prescribed to treat metabolic disorders in Asians. This study assessed whether GPs could prevent lithogenic diet (LD)-induced cholesterol gallstone (CG) formation and enhance CG dissolution in mice. Gallstone-susceptible C57BL/6J mice were fed an LD or normal chow, with or without GPs. Bile acids (BAs) in gallbladder bile were analyzed by liquid chromatography-tandem mass spectrometry. Differentially expressed hepatic genes were identified by RNA sequencing, followed by Gene Ontology (GO) and Kyoto Encyclopedia of Genes and Genomes (KEGG) pathway enrichment analyses. GPs were found to prevent LD-induced CG formation and to dissolve pre-existing LD-induced CGs. GPs reduced total cholesterol levels and increased BA levels in bile, as well as reducing the BA Hydrophobicity Index, ratio of 12α-hydroxylated (12α-OH) to non-12α-OH BAs, and Cholesterol Saturation Index in gallbladder bile. GO and KEGG pathway enrichment analyses indicated that GPs-induced genes were involved in BA biosynthesis and cholesterol metabolism. GPs increased the hepatic expression of genes encoding the cytochrome P450 (Cyp) enzymes Cyp7a1, Cyp7b1, and Cyp8b1, while decreasing the hepatic expression of genes encoding the adenosine triphosphate-binding cassette (Abc) transporters Abcg5 and Abcg8. GPs may be a promising strategy for preventing and dissolving CGs.

## Introduction

Cholelithiasis or gallstones are a prevalent and costly public health problem (1, 2). In Europe, ~20% of the adult population have gallstones, with cholesterol gallstones (CGs) accounting for more than 90% of all gallstones in Western populations (2, 3). Cholecystectomy currently represents the only effective treatment in the management of symptomatic gallbladder stones, but it has drawbacks, including risks of surgical complications and high economic costs (1, 2). Cholecystectomy can also shorten gut transit and induce the postcholecystectomy diarrhea syndrome (4). CGs can also be treated with the hydrophilic bile acid (BA) ursodeoxycholic acid (UDCA), although its clinical benefits are limited by difficulties in dose optimization, the risk of stone recurrence, and its high cost (3, 5). Novel approaches are therefore needed clinically for the prevention and treatment of CG disease.

CG formation results from a failure in biliary cholesterol homeostasis, involving a disturbance in the physical–chemical balance of cholesterol solubility in bile (3). Supersaturated bile is caused primarily by the hepatic hypersecretion of cholesterol and/or a deficiency in BAs or phospholipids (3). Cholesterol hypersaturation results in the precipitation of cholesterol crystals, which accumulate and form CGs (6). Biliary lipid secretion is regulated by an elaborate network of transporters. Cholesterol secretion is mediated by the adenosine triphosphate-binding cassette (Abc) transporters Abcg5 and Abcg8 (3), whereas Abcb4 is a major transporter of phospholipids (7). Bile acid export pump (Bsep) and multidrug resistance-associated protein 2 (Mrp2) are responsible for the secretion of BAs.

BA synthesis is the primary pathway for cholesterol catabolism (8), and increased hepatic BA synthesis can inhibit CG formation (9–12). The cytochrome P450 (Cyp) enzyme Cyp7a1 is the rate-limiting enzyme in the classical pathway, whereas Cyp27a1 is the first enzyme in the alternative pathway (8). Cyp8b1 and Cyp7b1 also play important roles in BA synthesis (8). Cyp2c70 is reported to catalyze the formation of muricholic acids (MCAs) (13). BAs are stored in the gallbladder temporarily and flow into the duodenum following food intake. BAs enable micelle formation and facilitate intestinal absorption, emulsification, and transport of lipid-soluble nutrients. Most BAs are reabsorbed by passive diffusion and active transport from the terminal ileum and are transported back to the liver *via* the portal vein (14), a process called the “enterohepatic circulation of BAs” (8).

**Figure 1:**
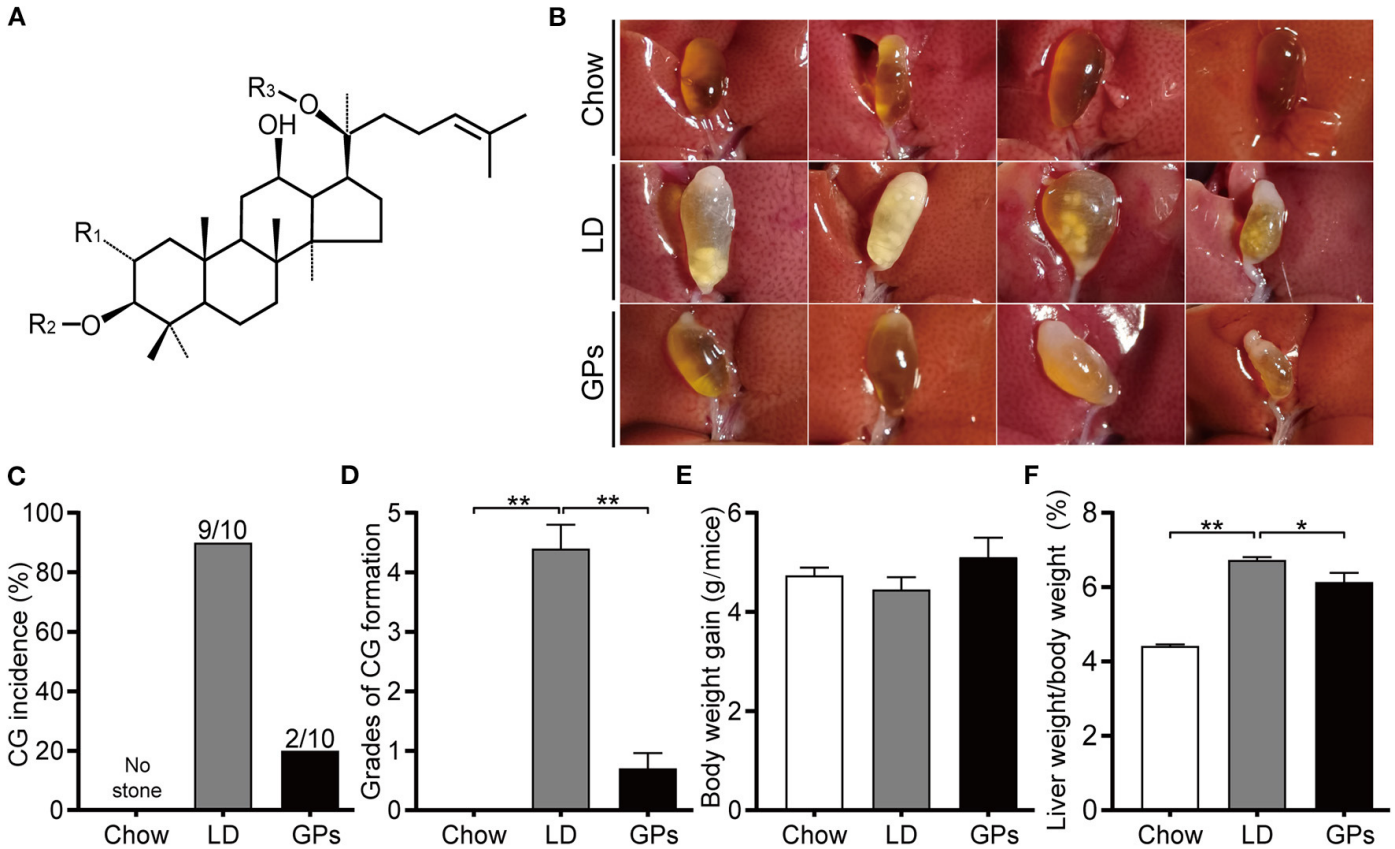
Effects of Gypenosides (GPs) on the prevention of cholesterol gallstones (CGs). **(A)** General structure of dammarane-type GPs. R1/R2 = glucose, rhamnose; R3 = glucose, xylose. **(B)** Representative gallbladders in each group. **(C)** CG incidence. **(D)** Grades of CG formation. **(E)** Body weight gain. **(F)** Ratio of liver weight-to-bodyweight. Data are reported as means ± SEM (*n* = 10). *P < 0.05, **P < 0.01.

The formation of CGs is profoundly influenced by metabolic abnormalities, such as dyslipidemia, diabetes mellitus and nonalcoholic fatty liver disease (3, 6). *Gynostemma pentaphyllum* (Thunb.) Makino (*G. pentaphyllum*), also known as “Jiao-Gu-Lan,” has a long history of use in China and several counties in East and Southeast Asia as folk remedies for many diseases, including hyperlipidemia, diabetes mellitus, and metabolic syndrome (15). Many products made from *G. pentaphyllum*, such as tea, instant powder, beverages, oral liquid, capsules, tablets, and pills, are commercially available in the United States and Europe, as well as in China and several other Asian countries (15). Gypenosides (GPs), the major ingredients of *G. pentaphyllum*, exist mainly as dammarane type-triterpene glycosides [(16); Figure 1A]. GPs were also reported to have a variety of pharmacological activities, including anti-hyperlipidemic, hepatoprotective, anti-inflammatory, and anti-diabetic properties (15, 17). It is unclear, however, whether GPs have beneficial effects in the prevention and dissolution of CGs. The present study therefore assessed the effects of GPs on mice with lithogenic diet (LD)-induced CGs and the mechanism of action of GPs.

## Materials and Methods

### Ethical Approval of the Study Protocol

All animal protocols were approved by the Animal Care and Use Committee of Shanghai Jiao Tong University Affiliated Sixth People's Hospital (Shanghai, China, Reference Number: DWSY2020-092).

### Animals and Treatment

Six-week-old male C57BL/6J mice (18–22 g) were purchased from Sipul-Bikai Laboratory Animal Co., Ltd. (Shanghai, China). Mice were maintained in a temperature-controlled room (20–24°C) with a 12:12-h light-dark cycle and were provided free access to water and designated food. The chow diet was LAD 0011, and the LD was TP 28900, both from Trophic Diet (Nantong, China). The LD contained 15% fat, 1.25% cholesterol and 0.5% cholic acid (CA). Mice were fed the chow diet or the LD, with or without GPs supplementation. GPs, derived from *Gynostemma pentaphyllum* (Thunb.) Makino, and of over 98% purity, was purchased from Xi'an Tian Guangyuan Biotech Co., Ltd. (Shanxi, China). The same batch of GPs was used in the present study.

To assess the ability of GPs to prevent CG formation, 30 mice were acclimated for 1 week on a chow diet and randomly separated into three groups of 10 mice each, housed five per cage. Mice in the Chow group were fed the normal chow diet, mice in the LD group were fed LD, and mice in the GPs group were fed LD supplemented with 2% GPs, all for 6 weeks. This dose was chosen because preliminary data showed that dietary supplementation with 2% GPs had a greater preventive effect than 1% or 5% GPs (Supplementary Figure 1).

To evaluate the ability of GPs to dissolve CGs, 30 mice were acclimated to a chow diet for 1 week, and subsequently fed the LD for 6 weeks, at which time > 90% of C57BL/6J mice had developed CGs (18). The 30 mice were randomly separated into three groups of 10 mice each, housed five per cage. Mice in the Vehicle group were fed the normal chow diet, mice in the low-dose GPs group (GPL) were fed the chow diet supplemented with 1% GPs, and mice in the high-dose GPs group (GPH) were fed the chow diet supplemented with 2% GPs, all for 4 weeks.

### Grades of CG Formation

The CG formation was graded as previously described (19). Briefly, the presence of crystals or gallstones was identified visually by holding the gallbladder up against a light. The scoring criteria are as follows: 0, gallbladder is filled with clear bile; 1, a few fine crystals are found; 2, around 10 fine crystals are found; 3, fine crystals occupy about a half of the gallbladder; 4, leaflet or stratified crystals occupy over a half of the gallbladder; 5, round gallstone are found.

### Analysis of Mouse Gallbladder Bile and Serum and Calculation of the Cholesterol Saturation Index

Concentrations of total cholesterol (TC) and BAs in gallbladder bile and concentrations of TC, triglycerides (TG), low-density lipoprotein-cholesterol (LDL-C) and high-density lipoprotein-cholesterol (HDL-C) in serum were measured enzymatically using kits obtained from the Jiancheng Bioengineering Institute (Nanjing, China). The concentrations of phospholipids in bile were measured using commercially available kits (Wako Pure Chemicals, Osaka, Japan). Gallbladder bile CSI was calculated as the actual molar percentage of TC in bile/highest concentration of soluble TC at a given bile molarity based on the Carey table (20).

### Analyses of Hepatic and Fecal Lipids and BA Composition in Gallbladder Bile

Hepatic and fecal lipids were extracted using a 2:1 (vol/vol) mixture of chloroform:methanol (18). The concentrations of TC and TG were measured using kits from Jiancheng Bioengineering Institute, as described. BA composition in gallbladder bile was measured by negative electrospray liquid chromatography-tandem mass spectrometry (LC-MS/MS) (18). Briefly, a 10-μL aliquot of bile was mixed with methanol, followed by a series of oscillations, centrifugations and dilutions with methanol. After filtration, supernatant was used for LC-MS/MS. The Hydrophobicity Index (HI) of gallbladder bile was calculated as reported previously (21, 22).

### RNA Sequencing and Functional Enrichment Analyses

Total RNA was extracted from mouse liver tissue using a tissue RNA purification kit (EZBioscience, Roseville, MN, USA), according to the manufacturer's instructions. RNA concentration was measured using Qubit 2.0 (Thermo Fisher Scientific) and RNA integrity was assessed using Agilent 2100 (Agilent Technologies). cDNA libraries were prepared according to standard Illumina RNA-seq instructions, and the libraries were sequenced on an Illumina NovaSeq 6000 platform to generate 150 bp paired-end reads. The raw reads of the fastq format were initially processed through primary quality control. Clean reads were obtained by removing low quality reads and contaminating adaptors. The high-quality reads were further aligned to the reference genome using HISAT2 (23). The number of reads mapped to each gene was determined by HTSeq (24). The log2-transformed fold changes in gene expression and significantly differentially expressed genes were calculated using edger (25). Differentially expressed genes were defined as those with a false discovery rate (FDR) < 0.1 and a log2 fold change > 1.

Kyoto Encyclopedia of Genes and Genomes (KEGG) and Gene Ontology (GO) pathway enrichment analyses of genes differentially expressed by the LD and GPs groups were performed using clusterProfiler (26). Significantly enriched KEGG pathways and GO terms of biological process, molecular function and cellular component categories were selected based on a Benjamini & Hochberg (BH) adjusted *p* < 0.05. The Gene-Concept Network depicts the linkages of genes and significantly enriched KEGG pathways and biological processes.

### Real-Time Reverse Transcription-Quantitative Polymerase Chain Reaction

Total RNA was extracted from liver and ileum tissues using a tissue RNA purification kit (EZBioscience, Roseville, MN, USA), according to the manufacturer's instructions. The quantity and purity of RNA was determined using a NanoDrop One spectrophotometer (Thermo Fisher Scientific, Waltham, MA, USA). RNAs were reverse transcribed using a HiScript™ III RT SuperMix for qPCR (Vazyme, Nanjing, China), according to the manufacturer's instructions. RT-qPCR was performed using AceQ Universal SYBR^®^ qPCR Master Mix (Vazyme), an ABI QuantStudio 7 Flex RT-PCR system (Applied Biosystems, Foster City, CA, USA), and the primers shown in Table 1. The levels of target gene mRNAs were quantified and normalized relative to the levels of glyceraldehyde 3-phosphate dehydrogenase (*Gapdh*) mRNA in the same samples.

**Table 1:** Primer sequences for quantitative real-time PCR analysis.

**Gene**	**Forward primer**	**Reverse primer**
*Cyp7b1*	GGAGCCACGACCCTAGATG	TGCCAAGATAAGGAAGCCAAC
*Cyp7a1*	AGCAGCCTCTGAAGAAGTGAATGG	AGAGCCGCAGAGCCTCCTTG
*Cyp8b1*	ACACCAAGGACAAGCAGCAAGAC	TGGCTCACTTCCACCCACTCC
*Cyp27a1*	ACACGGATGCCTTAAACGAGG	GCAGCCAATCCTTTTCTCAAAC
*Cyp2c70*	AGTATGGCCCTGTGTTTACTGT	GCCTTGGCTGGTTCTACTGAG
*β-klotho*	TGTTCTGCTGCGAGCTGTTAC	CCGGACTCACGTACTGTTTTT
*Mrp2*	GTGTGGATTCCCTTGGGCTTT	CACAACGAACACCTGCTTGG
*Mrp3*	CTGGGTCCCCTGCATCTAC	GCCGTCTTGAGCCTGGATAAC
*Mrp4*	GGCACTCCGGTTAAGTAACTC	TGTCACTTGGTCGAATTTGTTCA
*Oatp1a1*	GTGCATACCTAGCCAAATCACT	CCAGGCCCATAACCACACATC
*Oatp1a4*	GCTTTTCCAAGATCAAGGCATTT	CGTGGGGATACCGAATTGTCT
*Oatp1b2*	AGATCAGAGAAGACAAGGCACT	CTTTGGTCGGTGTAGCTTGG
*Bsep*	TCTGACTCAGTGATTCTTCGCA	CCCATAAACATCAGCCAGTTGT
*Asbt*	GTCTGTCCCCCAAATGCAACT	CACCCCATAGAAAACATCACCA
*Ntcp*	CAAACCTCAGAAGGACCAAACA	GTAGGAGGATTATTCCCGTTGTG
*Ostα*	AGGCAGGACTCATATCAAACTTG	TGAGGGCTATGTCCACTGGG
*Ostβ*	AGATGCGGCTCCTTGGAATTA	TGGCTGCTTCTTTCGATTTCTG
*Fgf15*	ATGGCGAGAAAGTGGAACGG	CTGACACAGACTGGGATTGCT
*Fgfr4*	GCTCGGAGGTAGAGGTCTTGT	CCACGCTGACTGGTAGGAA
*Abcb4*	CAGCGAGAAACGGAACAGCA	TCAGAGTATCGGAACAGTGTCA
*Fxr*	GCTTGATGTGCTACAAAAGCTG	CGTGGTGATGGTTGAATGTCC
*Shp*	TGGGTCCCAAGGAGTATGC	GCTCCAAGACTTCACACAGTG
*Abcg5*	AGGGCCTCACATCAACAGAG	GCTGACGCTGTAGGACACAT
*Abcg8*	CTGTGGAATGGGACTGTACTTC	GTTGGACTGACCACTGTAGGT
*Gapdh*	AGGTCGGTGTGAACGGATTTG	TGTAGACCATGTAGTTGAGGTCA

*Cyp7b1, cytochrome P450 7b1; Cyp7a1, cytochrome P450 7a1; Cyp8b1, cytochrome P450 8b1; Cyp27a1, cytochrome P450 27a1; Cyp2c70, cytochrome P450 2c70; β-klotho, beta-klotho; Mrp2, multidrug resistance-associated protein 2; Mrp3, multidrug resistance-associated protein 3; Mrp4, multidrug resistance-associated protein 4; Oatp1a1, organic anion-transporting polypeptide 1a1; Oatp1a4, organic anion-transporting polypeptide 1a4; Oatp1b2, organic anion-transporting polypeptide 1b2; Bsep, bile acid export pump; Asbt, apical sodium dependent bile acid transporter; Ntcp, Na/taurocholate cotransporting polypeptide; Ostα, organic solute transporter alpha; Ostβ, organic solute transporter beta; Fgf15, fibroblast growth factor 15; Fgfr4, fibroblast growth factor receptor 4; Abcb4, ATP-binding cassette, subfamily B, member 4; Fxr, bile acid receptor; Shp, nuclear receptor subfamily 0 group B member 2; Abcg5, ATP-binding cassette subfamily G member 5; Abcg8, ATP-binding cassette subfamily G member 8; Gapdh, glyceraldehyde-3-phosphate dehydrogenase*.

### Western Blotting

Proteins were extracted from liver tissue using RIPA lysis buffer (Beyotime Institute of Biotechnology, Shanghai, China), containing a protease and phosphatase inhibitor cocktail (Beyotime Institute of Biotechnology). Equal amounts of extracted proteins were separated by electrophoresis on 7.5% or 10% SDS-polyacrylamide gels and electrotransferred onto polyvinylidene fluoride membranes (Millipore, Tullagreen, Ireland). The membranes were blocked, incubated with appropriate primary antibodies at 4°C overnight, and subsequently incubated with secondary antibody (catalog number, 7074S, 7076S; Cell Signaling Technology, Danvers, MA, USA). Bands were detected using SuperSignal West Pico Chemiluminescent Substrate (Thermo Scientific) with a ChemiDoc MP imaging system (Bio-Rad Laboratories, Hercules, CA, USA).

The antibodies used, all of which were generated in rabbits and diluted 1:1000, were: anti-Gapdh (catalog number, 5174S; Cell Signaling Technology), anti-Abcg8 (DF6673; Affinity, Changzhou, China), anti-Cyp8b1 (ab191910; Abcam, Cambridge, UK), anti-Abcg5 (27722-1-AP; Proteintech, Rosemont, IL, USA), and anti-Cyp7b1 (24889-1-AP; Proteintech). Mouse anti-Cyp7a1 antibody (sc-518007; Santa Cruz Biotechnology, Dallas, TX, USA) was diluted 1:1500.

### Statistical Analyses

All data are expressed as means ± SEM. Differences between two groups were determined by two-tailed Student's *t*-test, whereas differences among three groups were determined by one-way analyses of variance (ANOVA) followed by Tukey's *post-hoc* test. All statistical analyses were performed using Prism version 8.0 (GraphPad Software), with *P* < 0.05 considered statistically significant.

## Results

### GPs Prevented LD-Induced CG Formation in Mice

Mice fed the chow diet had clear gallbladders, with no CGs, whereas 90% of mice in the LD group had “cloudy” gallbladders, containing cholesterol sludge and CGs (Figures 1B,C). In contrast, only 20% of mice in the GPs group developed cholesterol sludge or CGs. Grading of CG formation (19) showed that the grade was significantly higher in the LD than in the Chow group, but was reduced significantly upon GPs supplementation (Figure 1D). There were no significant differences in body weight gain among the three groups (Figure 1E). GPs were found, however, to partially reverse the LD-induced increase in liver weight-to-bodyweight ratio (Figure 1F).

### GPs Reduced the CSI of Gallbladder Bile and Ameliorated LD-Induced Lipid Metabolic Disorders in LD-Fed Mice

**Figure 2:**
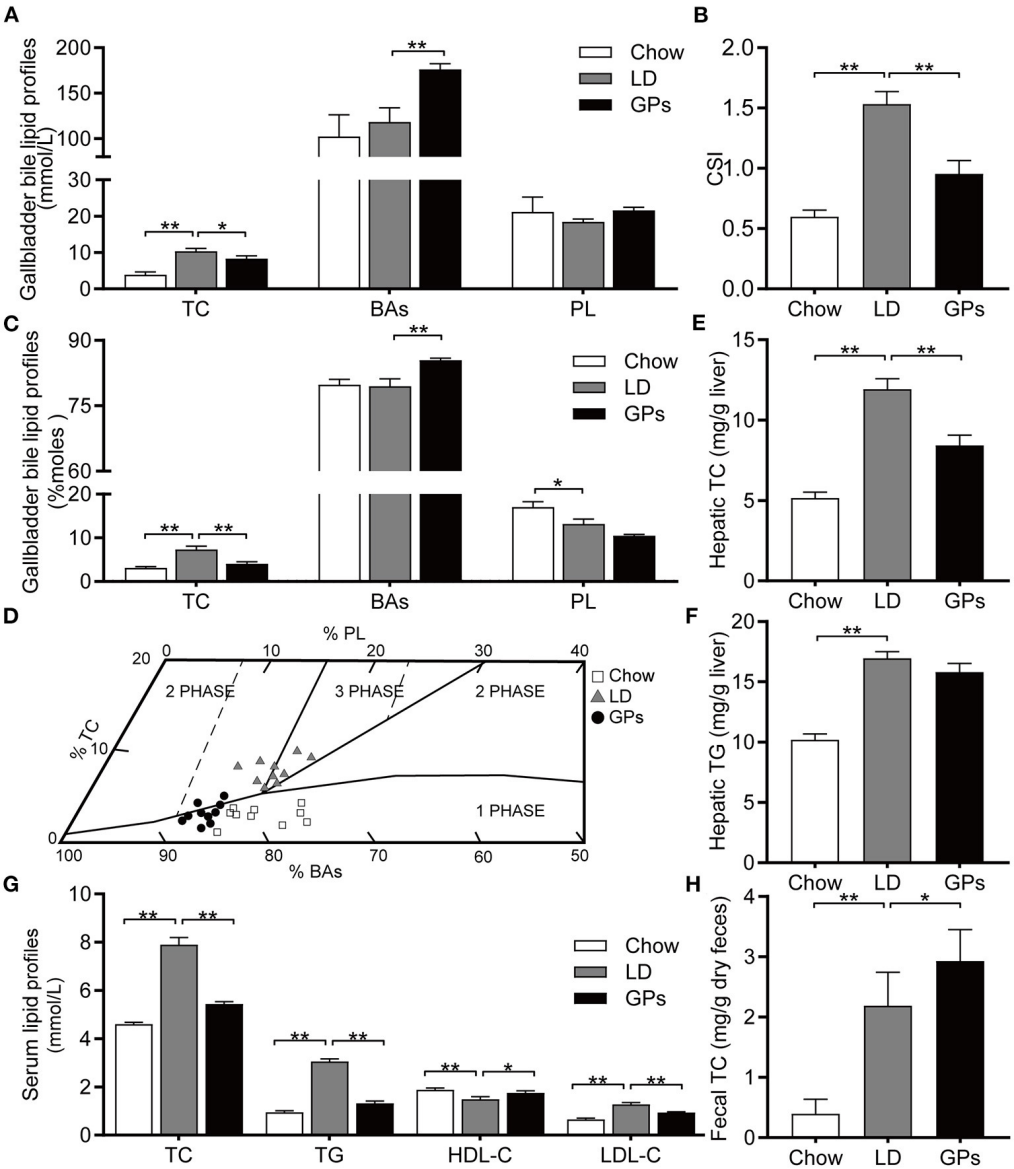
Effect of Gypenosides (GPs) on the lipid composition of gallbladder bile, and on lipid profiles in liver and serum. **(A)** Concentrations of total cholesterol (TC), bile acids (BAs) and phospholipids (PL). **(B)** Cholesterol Saturation Index (CSI). **(C)** Molar percentages of TC, BAs and PL. **(D)** Mean relative lipid composition (moles per 100 moles) of pooled gallbladder bile samples plotted on a condensed phase diagram. Depicted are the one-phase zone (only micelles); the left two-phase zone (containing micelles and crystals); the right two-phase zone (containing micelles and vesicles) and the three-phase zone (containing micelles, vesicles and crystals). **(E,F)** Hepatic concentrations of **(E)** TC and **(F)** triglycerides (TG). **(G)** Serum concentrations of TC, TG, low-density lipoprotein-cholesterol (LDL-C) and high-density lipoprotein-cholesterol (HDL-C). **(H)** Fecal concentration of TC. Data are reported as means ± SEM (*n* = 10). *P < 0.05, **P < 0.01.

Mice fed the LD without GPs showed high levels of TC and supersaturated bile (CSI > 1), correlating with the formation of CGs, whereas supplementation with GPs significantly reduced TC levels and the CSI of gallbladder bile (Figures 2A–C). GPs also significantly increased the level of BAs in gallbladder bile (Figures 2A,C). The relative lipid compositions of pooled gallbladder bile from mice fed the LD were located in the left 2-phase and 3-phase areas (Figure 2D), in which the bile was composed of cholesterol crystals and saturated micelles (6, 27). GPs supplementation, however, shifted the relative lipid compositions of pooled gallbladder bile downward and to the 1-phase area (Figure 2D). GPs significantly reduced hepatic TC levels, but did not significantly reduce hepatic TG levels (Figures 2E,F). GPs also significantly reduced the serum concentrations of TC, TG and LDL-C, and significantly increased the serum concentrations of HDL-C and fecal TC levels (Figures 2G,H).

### GPs Altered the BA Composition of Gallbladder Bile in LD-Fed Mice

**Figure 3:**
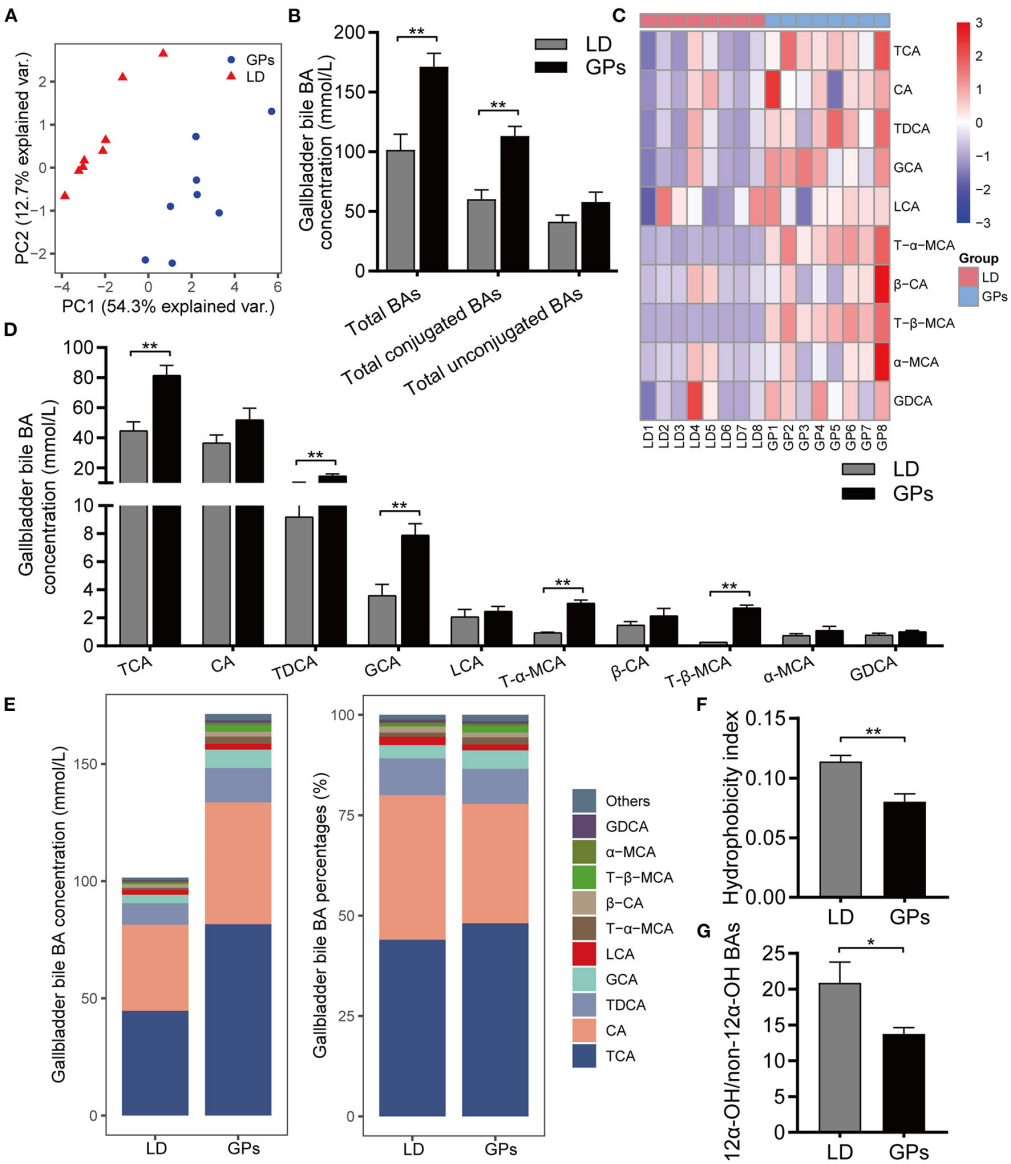
The bile acid (BA) composition of gallbladder bile. **(A)** Principal component analysis (PCA) of gallbladder bile. **(B)** Total BAs, conjugated BAs and unconjugated BAs in bile. **(C)** Heatmap of the top-10 most abundant BA species. **(D)** Concentrations of BAs. **(E)** Average concentration (left) and percentage (right) of individual BA species in each group. **(F)** Hydrophobicity Index of BAs. **(G)** Ratio of 12α-hydroxylated (12α-OH) BAs to non-12α-OH BAs. Data are reported as means ± SEM (*n* = 8). *P < 0.05, **P < 0.01.

In addition to the level of total BAs, the hydrophobic/hydrophilic profile of BAs has been associated with CG formation (6, 8, 28). We employed BA-targeted metabolomics to analyze the BA composition of gallbladder bile. Principal component analysis (PCA) showed obvious separation among groups (Figure 3A). GPs significantly increased the levels of total BAs and conjugated BAs in gallbladder bile, but did not significantly affect the levels of total unconjugated BAs (Figure 3B). GPs administration increased the levels of taurocholic acid (TCA), taurodeoxycholic acid (TDCA), glycocholic acid (GCA), tauro-β-muricholic acid (T-β-MCA), and T-α-MCA in gallbladder bile (Figures 3C–E). The mole percentages of individual BAs are shown in Supplementary Figure 2. GPs administration also reduced the HI of gallbladder bile BAs significantly (Figure 3F), as well as reducing the ratio of 12α-hydroxylated (12α-OH) BAs to non-12α-OH BAs (Figure 3G). These results indicated that GPs could alter the BA composition of gallbladder bile.

### GPs Altered Hepatic Cholesterol and BA Metabolic Gene Expression in LD-Fed Mice

**Figure 4:**
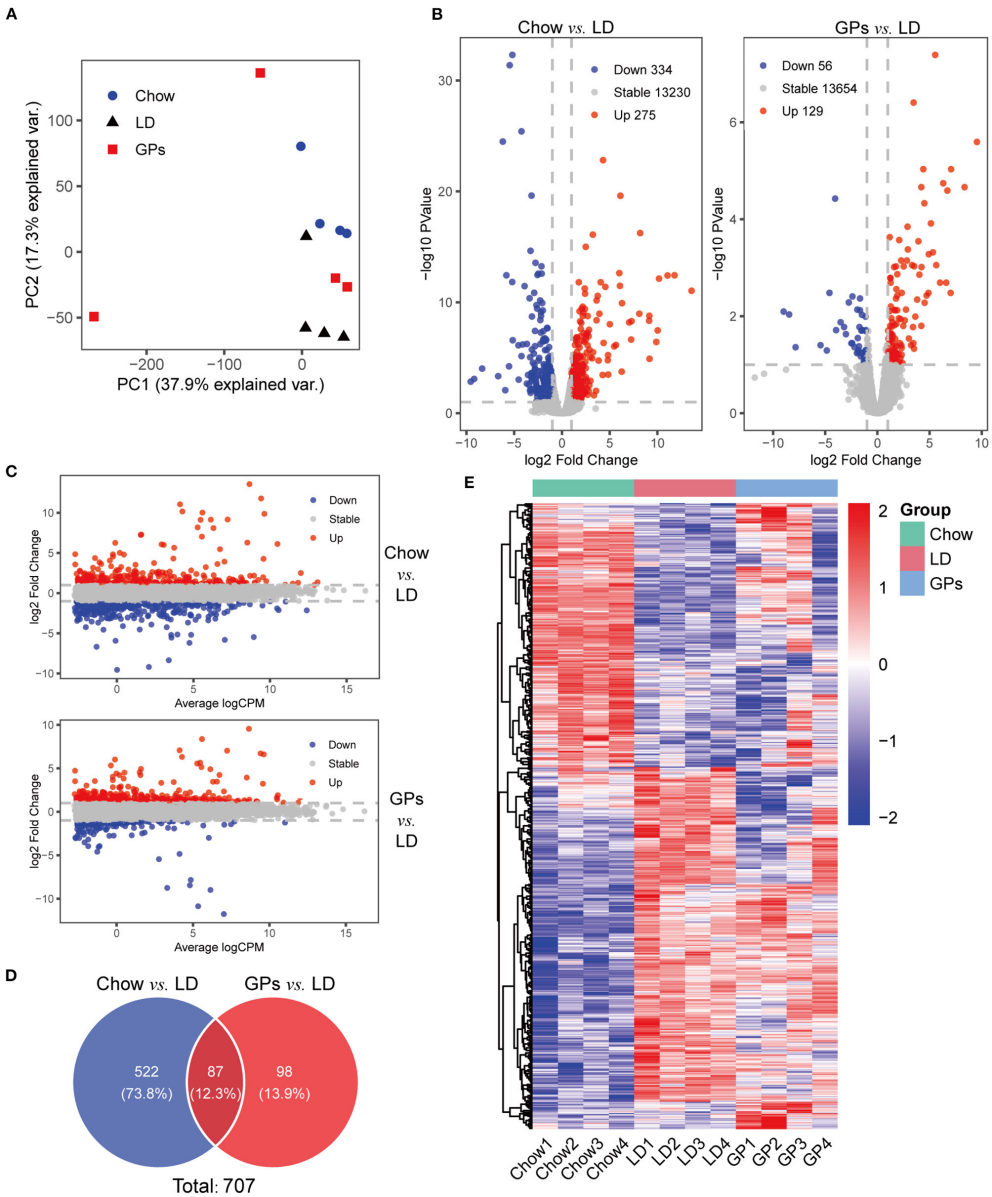
RNA sequencing (RNA-seq) analyses of liver tissue. **(A)** Principal component analysis (PCA) of the RNA-seq results. **(B)** Volcano plots of differentially expressed genes in the Chow and LD groups and in the GPs and LD groups (FDR < 0.1, log2 Fold Change > 1). **(C)** M-v-.A plots. **(D)** Venn diagrams. **(E)** Heatmap of the 707 differentially expressed genes from the two sets of comparisons. CPM, counts per million.

To explore the gene regulatory mechanisms involved in the prevention of CGs by GPs, RNA-seq analyses were performed using livers from the three groups of mice. PCA showed the separation among the three groups (Figure 4A). A comparison of the Chow and LD groups identified 609 differentially expressed genes with at least two-fold differences, including 275 genes that were up-regulated and 334 that were down-regulated (FDR < 0.1; Figures 4B,C and Supplementary Table 1). Subsequent comparison of the GPs and LD groups identified 185 differentially expressed genes with at least two-fold differences, including 129 genes that were up-regulated and 56 that were down-regulated (FDR < 0.1; Figures 4B,C and Supplementary Table 2). These two comparisons identified a total of 707 differentially expressed genes (Figure 4D). The heatmap of these 707 genes showed that GPs could alter hepatic gene expression in LD-fed mice (Figure 4E).

**Figure 5:**
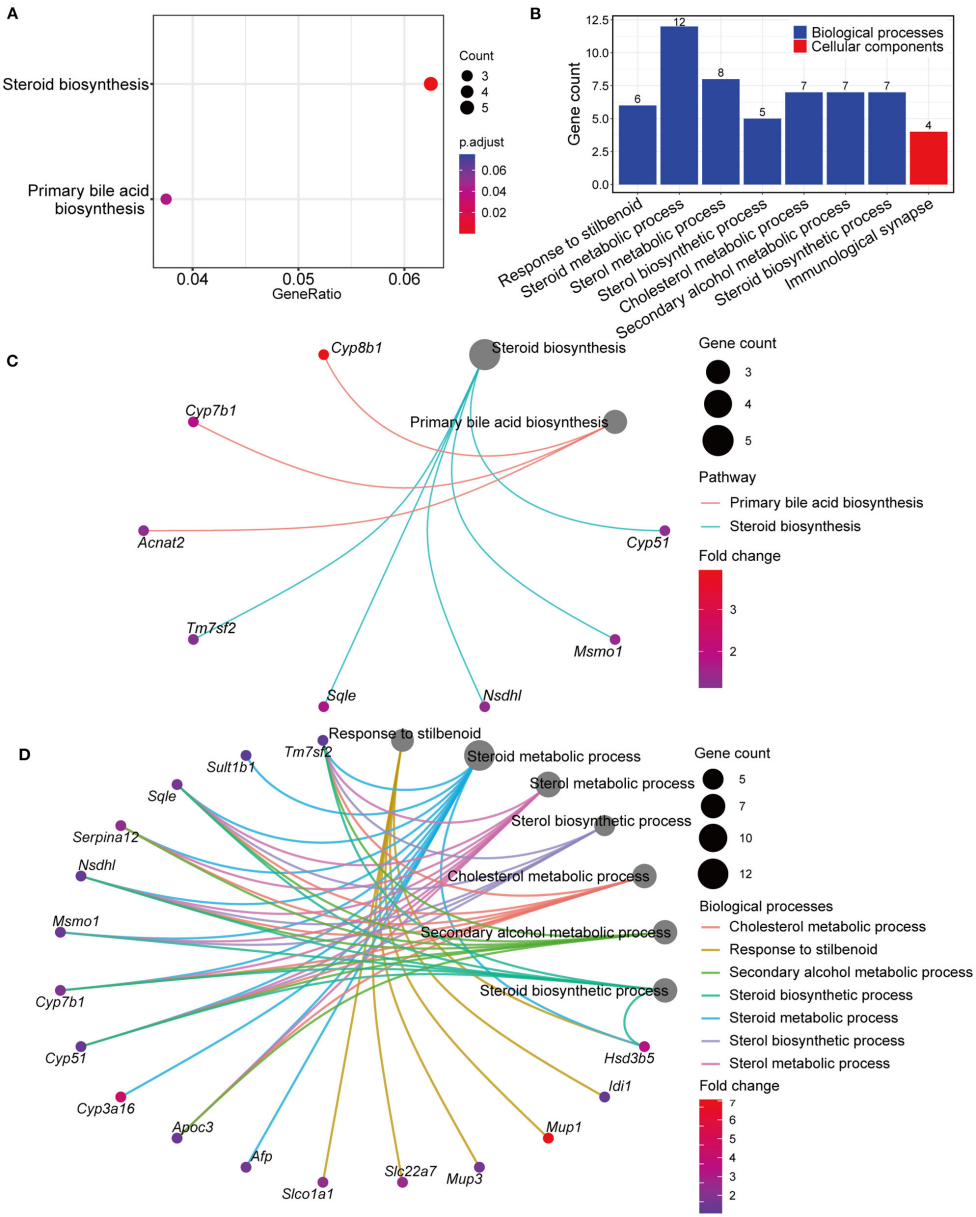
Kyoto Encyclopedia of Genes and Genomes (KEGG) and Gene Ontology (GO) pathway enrichment analyses of genes differentially expressed in the LD and GPs groups. **(A)** KEGG analyses (adjusted *p* < 0.05). **(B)** Significantly enriched biological processes and cellular components (adjusted *p* < 0.05). **(C)** Gene-Concept Network analysis, depicting the linkages of genes and significantly enriched KEGG pathways. **(D)** Gene-Concept Network analysis of genes and significantly enriched biological processes.

Genes differentially expressed in the GPs and LD groups were further analyzed by KEGG and GO pathway enrichment analyses. Interestingly, KEGG analyses showed that the differentially expressed genes included those involved in steroid and BA biosynthesis (Figure 5A and Supplementary Table 3). GO analyses showed that the differentially expressed genes included those involved in biological processes, such as steroid, sterol and cholesterol metabolism (Figure 5B and Supplementary Table 4). The Gene-Concept Network showed the linkages of genes and significantly enriched KEGG pathways and biological processes (Figures 5C,D). These results indicated that GPs may inhibit CG formation by modulating BA synthesis and cholesterol metabolism.

### GPs Enhanced BA Synthesis and Decreased the Canalicular Efflux of Cholesterol in LD-Fed Mice

**Figure 6:**
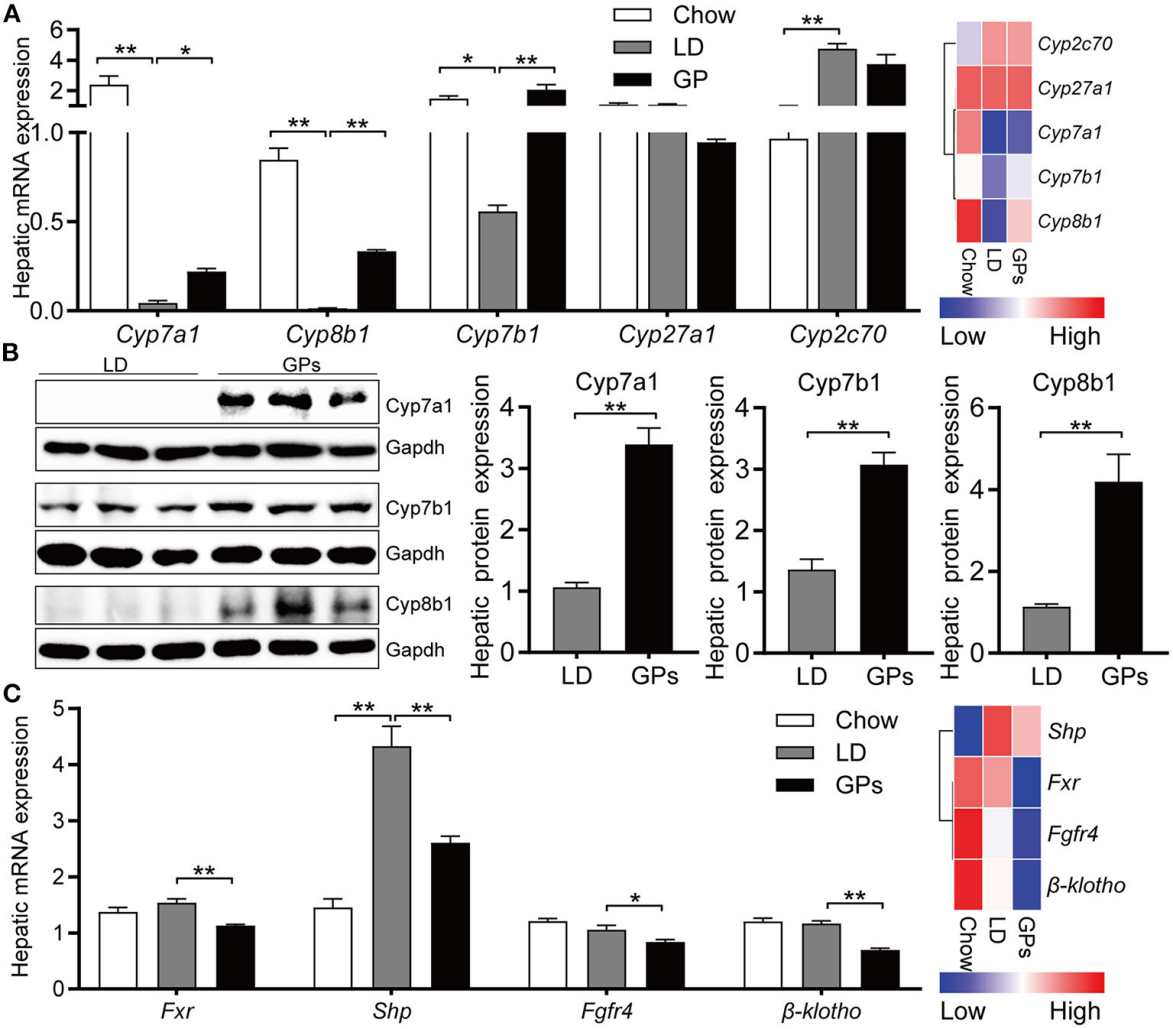
Hepatic expression of genes involved in bile acid synthesis and regulation. **(A)** Levels of expression of *Cyp7a1, Cyp8b1, Cyp7b1, Cyp27a1*, and *Cyp2c70* mRNAs measured by qRT-PCR (left panel, *n* = 8) and RNA sequencing (RNA-seq) analyses (right panel, data shown as means). **(B)** Protein expression and quantification of Cyp7a1, Cyp781, and Cyp7b1 (*n* = 6). **(C)** Levels of expression of *Fxr, Shp, Fgfr4* and β*-klotho* mRNAs measured by qRT-PCR (left panel, *n* = 8) and RNA-seq analyses (right panel, data shown as means). Data are reported as means ± SEM. *P < 0.05, **P < 0.01.

To further confirm the inhibitory effect of GPs on the CG formation by modulating BA and cholesterol metabolism, we assessed the expression of genes involved in cholesterol and BA metabolism. Hepatic expression of *Cyp7a1, Cyp7b1*, and *Cyp8b1* mRNAs was decreased significantly in mice fed LD, whereas GPs significantly reverse these reductions (Figure 6A). GPs did not significantly alter the hepatic expression of *Cyp2c70* mRNAs (Figure 6A). Western blotting analyses also showed that the protein levels of Cyp7a1, Cyp7b1, and Cyp8b1 were increased by GPs (Figure 6B). GPs also significantly decreased the hepatic expression of mRNAs encoding farnesoid X receptor *(Fxr)*, small heterodimer partner *(Shp)*, fibroblast growth factor receptor 4 (*Fgfr4*) and β*-klotho* (Figure 6C), all of which are associated with the regulation of BA synthesis.

**Figure 7:**
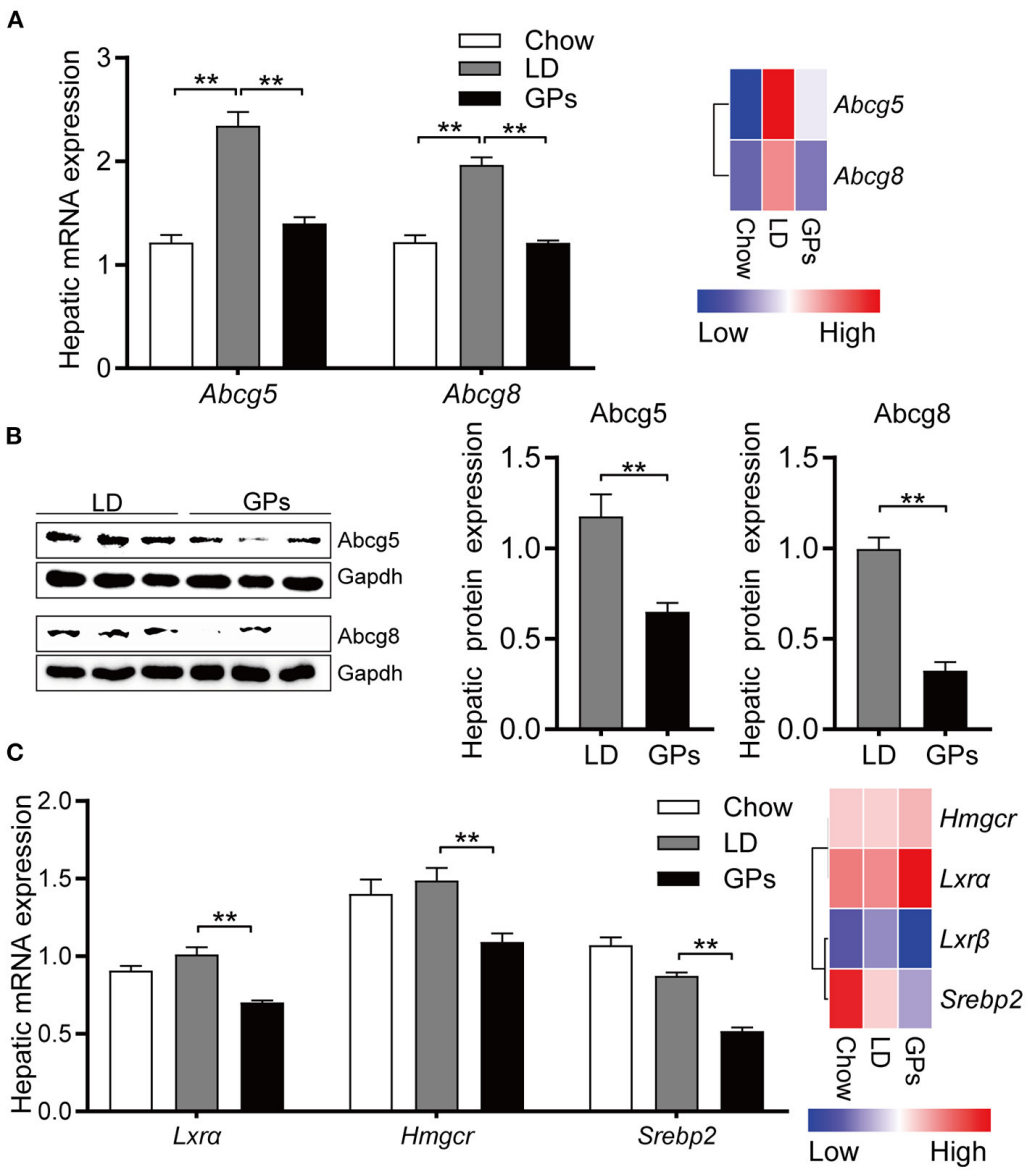
Hepatic expression of genes involved in cholesterol transport and synthesis. **(A)** Levels of expression of Abcg5 and Abcg8 mRNAs measured by qRT-PCR (left panel, *n* = 8) and RNA sequencing (RNA-seq) analyses (right panel, data shown as means). **(B)** Protein expression and quantification of Abcg5 and Abcg8 (*n* = 6). **(C)** Levels of expression of Lxrα, Hmgcr and Srebp2 mRNAs measured by qRT-PCR (left panel, *n* = 8) and RNA-seq analyses (right panel, data shown as means). Data are reported as means ± SEM. **P < 0.01.

Consistent with the reduced cholesterol level in bile (Figure 2A), GPs significantly reduced the hepatic mRNA and protein expression of *Abcg5* and *Abcg8* (Figures 7A,B). The levels of mRNA encoding 3-hydroxy-methylglutaryl CoA reductase (*Hmgcr*) and sterol regulatory element-binding protein 2 (*Srebp2*) were also reduced in GPs-treated mice (Figure 7C). Together, these results indicated that GPs could enhance cholesterol catabolism by increasing *de novo* synthesis of BAs and reduce the LD-induced hepatic hypersecretion of cholesterol.

### GPs Altered the Expression of Genes Involved in BA Transport in the Liver and Ileum

Enterohepatic circulation of BAs is mediated by a series of transporters in the liver and ileum. Organic anion-transporting polypeptides (Oatps) and sodium taurocholate cotransporting polypeptide (Ntcp) mediate the uptake of BAs from portal blood into hepatocytes (29) GPs treatment significantly increased the expression of *Oatp1a1* mRNA and significantly reduced the expression of *Oatp1a4* mRNA ((Supplementary Figure 3). Assessment of mRNAs encoding efflux transporters in the sinusoidal membrane of hepatocytes showed that the LD-induced increases in *Mrp3* and *Mrp4* mRNA levels were significantly reduced by GPs (Supplementary Figure 3). Bsep and Mrp2 mediate the secretion of BAs from hepatocytes into the bile (29). GPs decreased the expression of *Mrp2* mRNA, but had little effect on the expression of *Bsep* mRNA (Supplementary Figure 3A). GPs also significantly reduced the expression of *Abcb4* mRNA (Supplementary Figure 3C).

Apical sodium-dependent bile acid transporter (Asbt) and organic solute transporter α/β (Ostα/β) are responsible for the reabsorption of BAs in the ileum. GPs significantly increased the expression of *Asbt* mRNA, but had little effect on the expression of *Ost*α*/*β mRNA (Supplementary Figure 4A). LD significantly enhanced the expression of fibroblast growth factor 15 (*Fgf15*) and *Shp* mRNAs in the ileum, but these changes were significantly reversed by GPs (Supplementary Figure 4C). Niemann-Pick C1-like 1 (Npc1l1) mediates cholesterol uptake and the Abcg5/g8 heterodimer is responsible for the transintestinal excretion of cholesterol (30). LD consumption significantly reduced the level of *Npc1l1* mRNA, perhaps through the activation of *Shp* (31), whereas GPs supplementation significantly increased the expression of *Npc1l1* mRNA (Supplementary Figure 4). GPs treatment also significantly enhanced the levels of liver X receptor α (*Lxr*α), *Abcg5* and *Abcg8* mRNAs (Supplementary Figure 4C).

### GPs Dissolved LD-Induced Preexisting CGs in Mice

**Figure 8:**
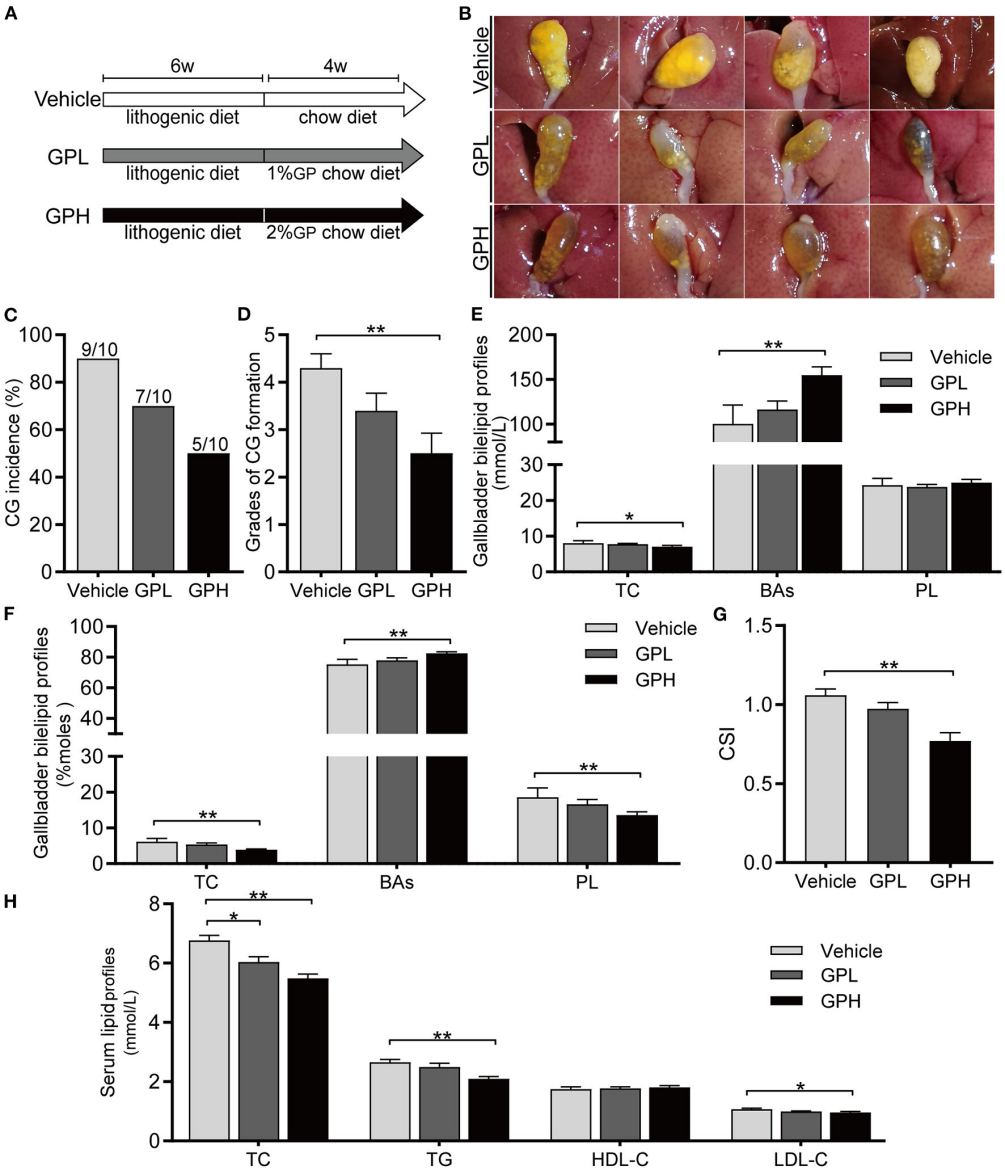
Effect of GPs on the dissolution of preexisting cholesterol gallstones (CGs). **(A)** Study design showing diets and durations of GPs treatments. **(B)** Representative gallbladders per group. **(C)** CG incidence. **(D)** Grades of CG formation. **(E)** Bile concentrations of total cholesterol (TC), bile acids (BAs) and phospholipids (PL). **(F)** Molar percentages of TC, BAs and PL. **(G)** Cholesterol Saturation Index (CSI). **(H)** Serum concentrations of TC, triglycerides (TG), low-density lipoprotein-cholesterol (LDL-C) and high-density lipoprotein-cholesterol (HDL-C). Data are reported as means ± SEM (*n* = 10). *P < 0.05, **P < 0.01.

To determine whether GPs can dissolve preexisting CGs, mice were fed LD for 6 weeks and then changed to a Chow diet with or without GPs supplementation for 4 weeks (Figure 8A). CGs were present in 90% of mice changed to a normal chow diet without GPs supplementation (Figures 8B,C), indicating that CGs did not dissolve spontaneously after the LD was replaced by the chow diet for 4 weeks. In contrast, GPs supplementation reduced the incidence of CGs in mice, with CGs being present in 70% and 50% of mice in the GPL and GPH groups, respectively (Figures 8B,C). GPs supplementation also significantly reduced the grade of CGs (Figure 8D). GPs significantly reduced the TC level and increased the BA level in gallbladder bile (Figures 8E,F), thereby reducing the CSI of bile (Figure 8G). GPs also significantly reduced the serum levels of TC, TG and LDL-C (Figure 8H). These results indicated that GPs could dissolved LD-induced preexisting CGs partially.

## Discussion

The present study showed that GPs could both prevent the development of CGs and dissolve LD-induced CGs in mice. GPs reduced the level of TC and increased the level of BAs in bile. GPs also decreased the HI of BAs, the ratio of 12α-OH to non-12α-OH BAs, and CSI in bile. KEGG and GO pathway enrichment analyses indicated that GPs might inhibit CG formation by modulating BA biosynthesis and cholesterol metabolism.

CG disease is associated with several metabolic abnormalities, including obesity, dyslipidemia, atherosclerosis, type-2 diabetes mellitus, and nonalcoholic fatty liver disease (3, 6). *G. pentaphyllum* is widely used in Asian countries as an herbal tea, dietary supplement, vegetable, and herb. GPs, the major ingredients of *G. pentaphyllum*, have been reported to ameliorate obesity, hyperlipidemia, hepatic steatosis, and insulin resistance (15, 17, 32, 33). GPs treatment has been approved in China as a safe and low-cost treatment for hyperlipidemia. The present results indicated that GPs could prevent LD-induced CG formation in a murine model of CGs. Additionally, GPs ameliorated LD-induced hypercholesterolemia and hypertriglyceridemia.

The major factor in CG formation is cholesterol hypersaturation in bile (3). Excess amounts of cholesterol in supersaturated bile cannot be solubilized by BAs and phospholipids. We found that GPs alleviated the supersaturated state of bile by reducing the levels of TC and increasing the levels of BAs in gallbladder bile. Alterations in BA composition also influence cholesterol solubilization and/or precipitation in bile (6). Hydrophilic BAs, such as MCA and UDCA, can promote hepatic BA efflux and bile flow and have beneficial effects on the prevention and dissolution of CGs (6, 8, 34, 35). Hydrophilic bile acids can inhibit intestinal cholesterol absorption (36). We found that GPs increased the level of hydrophilic BAs, including T-β-MCA and T-α-MCA, in gallbladder bile, thereby reducing the HI of BAs in bile, which could increase the solubility of biliary cholesterol. GPs also increased the fecal TC level. An increased ratio of 12α-OH to non-12α-OH BAs has been associated with metabolic diseases (37), with a reduction in this ratio playing a role in controlling lipid absorption (38). We found that GPs favored increased production of non-12α-OH BAs and improved metabolic phenotypes.

BA synthesis is a major pathway for cholesterol catabolism and important for maintenance of whole-body cholesterol homeostasis, with Fxr being a key regulator of BA synthesis (8). Activation of hepatic Fxr inhibits BA synthesis by up-regulating the expression of Shp (39). In the ileum, activation of Fxr induces the expression of Fgf15 (FGF19 in humans), which is secreted into the portal vein and transported to the liver, where it binds to the Fgfr4/β-klotho heterodimer complex to inhibit hepatic BA synthesis (39). The CA in LD can activate Fxr and inhibit BA synthesis (40). Increased hepatic BA synthesis has been shown to inhibit CG formation (9–12). In this study, RNA seq analyses showed that GPs could alter significantly alter the hepatic expression of cholesterol and BA genes in LD-fed mice. Furthermore, KEGG and GO pathway enrichment analyses showed that genes differentially expressed in the GPs group relative to the LD were strongly enriched in the BA biosynthesis and cholesterol metabolism pathways. GPs have been reported to enhance BA synthesis from cholesterol (41). We found that GPs increased the hepatic expression of the enzymes Cyp7a1, Cyp7b1, and Cyp8b1, which enhanced BA synthesis and cholesterol catabolism. Consistent with these findings, the levels of cholesterol in the bile and liver were lower, as was the hepatic expression of the Abcg5/g8 heterodimer, a transporter of biliary cholesterol secretion (3). Increased hepatic BA synthesis also altered the BA composition and increased the level of hydrophilic BAs in bile. Increased BA synthesis may be associated with reduced expression of Fgf15 in the ileum and Shp in the liver, but this requires further verification.

GPs have been reported to inhibit pancreatic lipase activity and reduce intestinal cholesterol absorption by inhibiting micelle formation *in vitro* (42). We found that GPs decreased the hepatic expression of Hmgcr and increased the ileal expression of Abcg5 and Abcg8, indicating that GPs could inhibit cholesterol synthesis and increase the transintestinal excretion of cholesterol. Additional studies, however, are required to assess the effects of GPs on the synthesis and transintestinal excretion of cholesterol.

Replacement of a lithogenic diet with a chow diet did not result in the spontaneous dissolution of gallstones (5). Similarly, the present study found that CGs were still present in 90% of mice after their diet was changed from an LD to a normal chow diet. GPs supplementation could partially dissolve the LD-induced preexisting CGs in mice and alleviate the supersaturated state of bile, along with reducing levels of TC and increasing levels of BAs in gallbladder bile.

Mouse models of CGs with cholesterol and CA were first reported in 1964 (43) and have since been used widely (5, 12, 28, 44–46). However, the relative compositions of BA pools differ in humans and mice (39). Further studies are needed to accelerate translation from mice to humans. At present, the effects of GPs on CGs in humans remain unknown. We are now carrying out the clinical trials to explore whether GPs can alter the biliary lipid composition in patients and act as a biliary cholesterol-desaturating agent.

## Conclusions

Although cholecystectomy is currently the only effective treatment in the management of symptomatic gallbladder stones, it has several drawbacks, including risks of surgical complications, high economic costs and postcholecystectomy diarrhea syndrome (1, 2, 4). Finding novel drugs for the prevention and treatment of CG disease has important clinical significance. In conclusion, we found that GPs, which are safe and low-cost, could ameliorate CG disease in mice, perhaps by increasing hepatic BA synthesis. Our findings suggest a novel strategy for the prevention and dissolution of CGs.

## Data Availability Statement

The datasets presented in this study can be found in online repositories. The names of the repository/repositories and accession number(s) can be found at NCBI BioProject, accession number, PRJNA771918.

## Ethics Statement

The animal study was reviewed and approved by the Animal Care and Use Committee of Sixth People's Hospital Affiliated to Shanghai Jiao Tong University.

## Author Contributions

QZ and JC conceived the study, performed most of the experiments, and wrote the manuscript. JX, MN, and SW helped with the experiments. SS, YS, and DH helped perform the data analyses. ZD and XW supervised the research and revised the manuscript. All authors contributed to the article and approved the submitted version.

## Funding

This work was supported by the National Natural Science Foundation of China (81870452) and grants from the Shanghai Science and Technology Program (19411951500 and 20JC1419302).

## Conflict of Interest

The authors declare that the research was conducted in the absence of any commercial or financial relationships that could be construed as a potential conflict of interest.

## Publisher's Note

All claims expressed in this article are solely those of the authors and do not necessarily represent those of their affiliated organizations, or those of the publisher, the editors and the reviewers. Any product that may be evaluated in this article, or claim that may be made by its manufacturer, is not guaranteed or endorsed by the publisher.

## Supplementary Material

Supplementary Figure 1 Inhibitory effects of 1% GPs and 5% GPs on LD-induced cholesterol gallstone (CG) formation in mice. **(A)** Representative gallbladders per group. **(B)** CG incidence. **(C)** Grades of CG formation. Data are reported as means 1 SEM (*n* = 10). ^*^*P* < 0.05, ^**^*P* < 0.01.

Supplementary Figure 2 Percentage of bile acids in gallbladder bile. Data are reported as means ± SEM (*n* = 8). ^*^*P* < 0.05, ^**^*P* < 0.01.

Supplementary Figure 3 Hepatic expression of genes involved in bile acid (BA) transport. **(A,B)** mRNA expression of genes involved in BA transport, as measured by **(A)** qRT-PCR (n = 8) and **(B)** RNA sequencing analyses (data shown as means). **(C)** Expression of Abcb4 mRNA (*n* = 8). Data are reported as means ± SEM. ^*^*P* < 0.05, ^**^*P* < 0.01.

Supplementary Figure 4 Ileal expression of genes involved in bile acid (BA) and cholesterol transport. Levels of expression of **(A)** Npc1l1, Abcg5, Abcg8 and Lxrα mRNAs, of **(B)** Asbt and Ostα/β mRNAs, and of **(C)** Fxr, Shp and Fgf15 mRNAs. Data are reported as means ± SEM (*n* = 8). ^*^*P* < 0.05, ^**^*P* < 0.01.

Supplementary Table 1 Differentially expressed genes in the Chow and LD groups.

Supplementary Table 2 Differentially expressed genes in the GPs and LD groups.

Supplementary Table 3 Kyoto Encyclopedia of Genes and Genomes (KEGG) pathway enrichment analyses of genes differentially expressed in the LD and GPs groups.

Supplementary Table 4 Gene Ontology (GO) pathway enrichment analyses of genes differentially expressed in the LD and GPs groups.
