# Supplementary figures and images for: Gypenosides Prevent and Dissolve Cholesterol Gallstones by Modulating the Homeostasis of Cholesterol and Bile Acids
Source: Front Med (Lausanne). 2022 Apr 4;9:818144. doi: 10.3389/fmed.2022.818144 (PMC9013900; doi:10.3389/fmed.2022.818144)

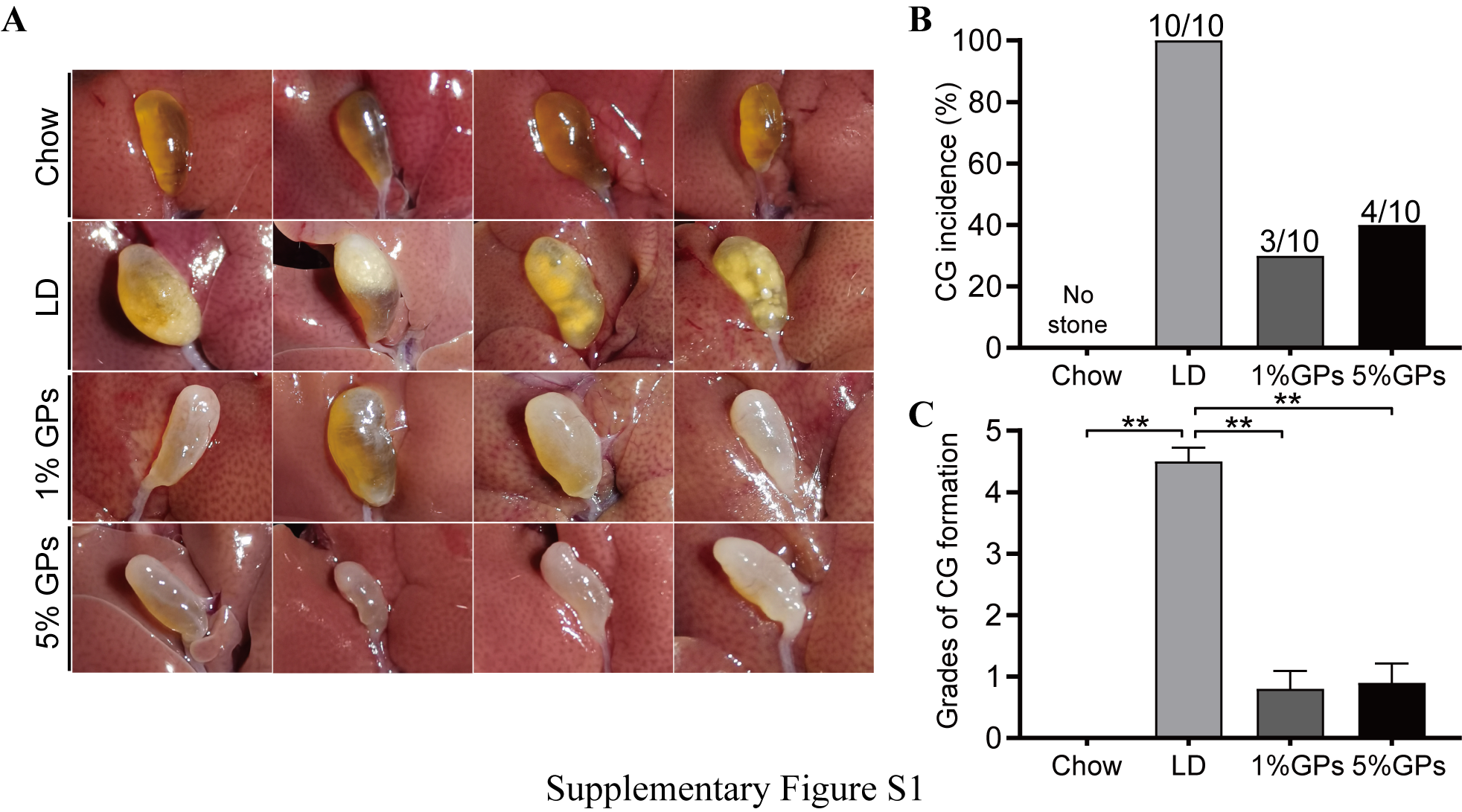

Supplement: Supplementary Figure 1 — Inhibitory effects of 1% GPs and 5% GPs on LD-induced cholesterol gallstone (CG) formation in mice. (A) Representative gallbladders per group. (B) CG incidence. (C) Grades of CG formation. Data are reported as means 1 SEM (n = 10). *P < 0.05, **P < 0.01. [file Image_1.tif]

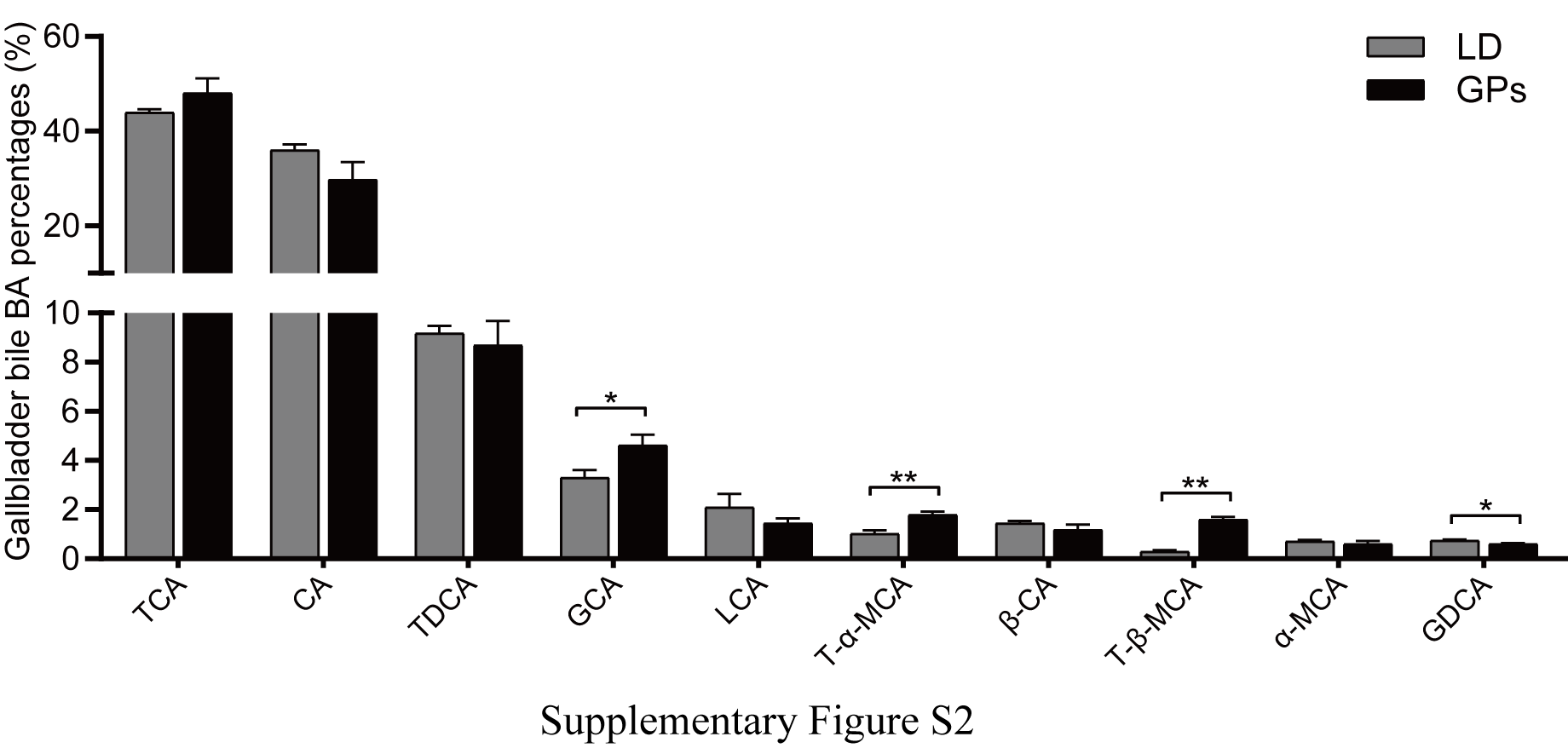

Supplement: Supplementary Figure 2 — Percentage of bile acids in gallbladder bile. Data are reported as means ± SEM (n = 8). *P < 0.05, **P < 0.01. [file Image_2.tif]

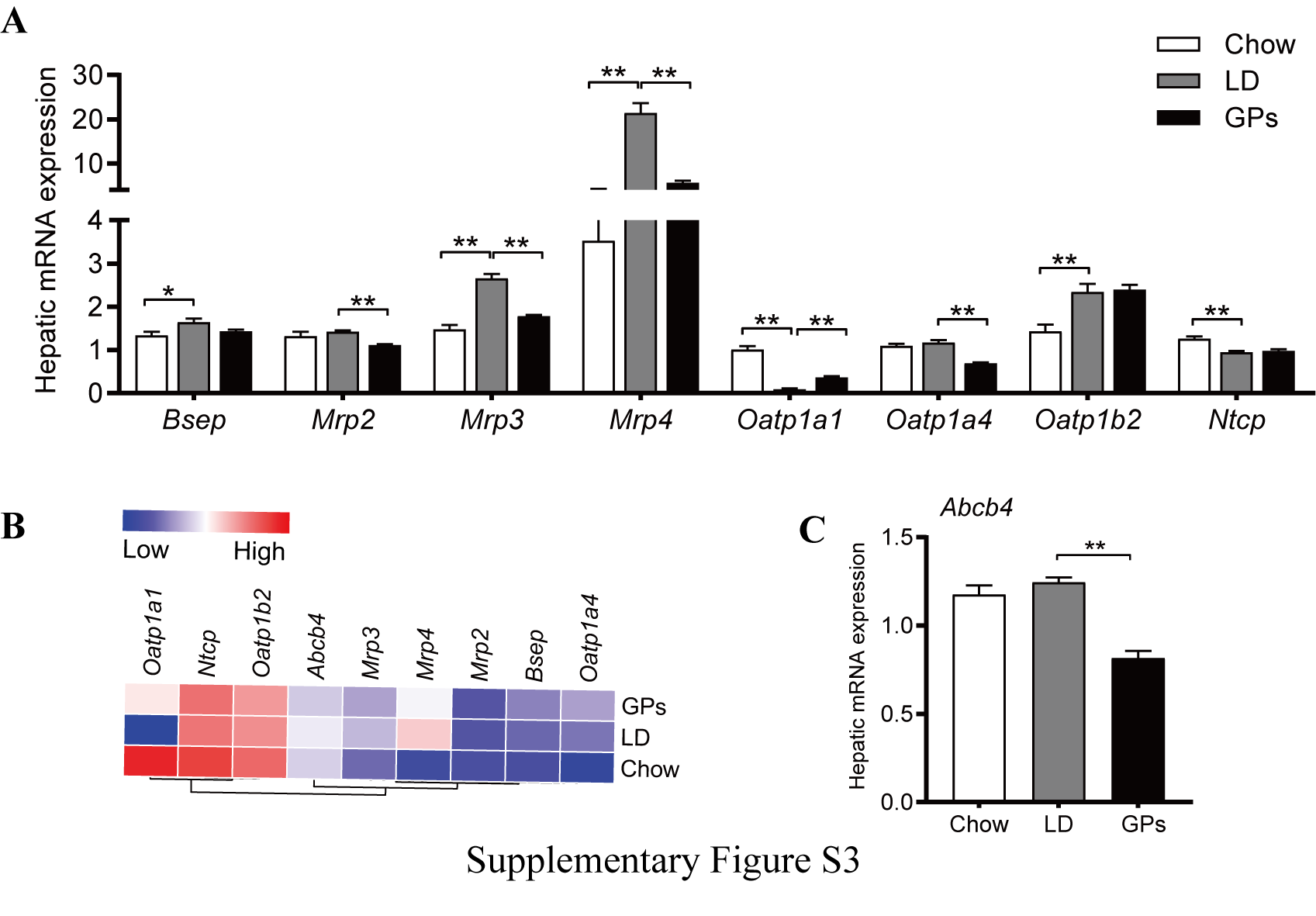

Supplement: Supplementary Figure 3 — Hepatic expression of genes involved in bile acid (BA) transport. (A,B) mRNA expression of genes involved in BA transport, as measured by (A) qRT-PCR (n = 8) and (B) RNA sequencing analyses (data shown as means). (C) Expression of Abcb4 mRNA (n = 8). Data are reported as means ± SEM. *P < 0.05, **P < 0.01. [file Image_3.tif]

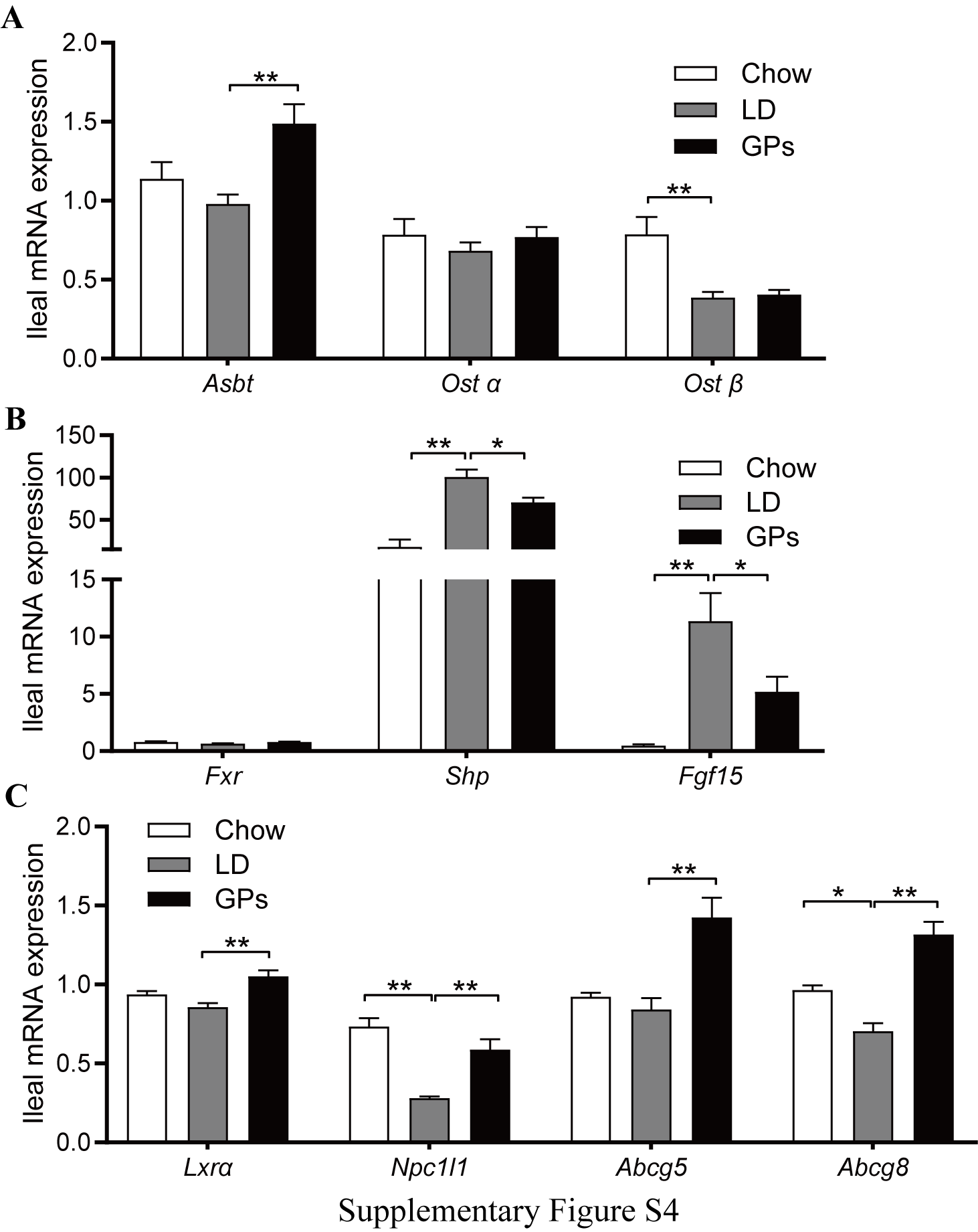

Supplement: Supplementary Figure 4 — Ileal expression of genes involved in bile acid (BA) and cholesterol transport. Levels of expression of (A) Npc1l1, Abcg5, Abcg8 and Lxrα mRNAs, of (B) Asbt and Ostα/β mRNAs, and of (C) Fxr, Shp and Fgf15 mRNAs. Data are reported as means ± SEM (n = 8). *P < 0.05, **P < 0.01. [file Image_4.tif]
